# Correction to: Abhishek et al., Antioxidant and anti-inflammatory activities of *Aerva pseudotomentosa* leaves

**DOI:** 10.1080/13880209.2017.1328825

**Published:** 2017-05-16

Abhishek P, Atul K, Manish W, Yadu ND, Bhagat SJ, Anamika D, et al. 2017. Antioxidant and anti-inflammatory activities of *Aerva pseudotomentosa* leaves. Pharmaceutical Biology. 55:1688–1697.

https://doi.org/10.1080/13880209.2017.1321022

When the above article was first published online, there were some errors in the text which have now been corrected in both online and print version.

